# Valorization of Banana Peel Using Carbonization: Potential Use in the Sustainable Manufacturing of Flexible Supercapacitors

**DOI:** 10.3390/mi14020330

**Published:** 2023-01-27

**Authors:** Melkie Getnet Tadesse, Esubalew Kasaw, Jörn Felix Lübben

**Affiliations:** 1 Sustainable Engineering (STE), Albstadt-Sigmaringen University, 72458 Albstadt, Germany; 2 Ethiopian Institute of Textile and Fashion Technology, Bahir Dar University, Bahir Dar 1037, Ethiopia

**Keywords:** activated banana peel, supercapacitor, bio-waste, BET-surface, XRD, CV

## Abstract

Sustainable and environmentally friendly activated carbon from biomass materials is proposed to produce supercapacitors from banana peels and has the potential to replace the non-sustainable and hazardous process from either graphite or/and fossil fuels. In order to determine the potential of using banana peel for supercapacitor application, raw banana peel, a bio-waste, was activated both mechanically and chemically to observe the real differences. The sample was activated at 700 °C and chemically activated using KOH. Characterization of activated banana peel was performed using FTIR, DLS, TGA and XRD analytical equipment. FTIR analysis revised the presence of hydroxyl, carbonyl and aromatic compounds on a banana peel cellulose-based carbon. The TGA results proved that 700 °C could be sufficient to totally carbonize banana peel. DLS clearly showed a strong difference between the carbonized and KOH-activated material in particle size distribution. Meanwhile, surface area analysis using BET displayed an increase from 553.862 m^2^/g to 565.024 m^2^/g BET in surface area (SBET) when carbon was activated using KOH with a nitrogen isotherm at 77.350 K. Specific capacitance was increased from 0.3997 Fg^−1^ to 0.821 Fg^−1^, suggesting more than a 100% increase in the specific capacity due to KOH activation, as proved by the cyclic voltammetry (CV) curve. The X-ray diffraction results revealed the patterns of activated carbon. The findings demonstrated the feasibility of using banana peel waste as a low-cost and sustainable material for the preparation of flexible supercapacitor batteries.

## 1. Introduction

Supercapacitors have received significant attention in recent years. The majority of studies have focused on improving their lifecycle [1], size [2], power density [3], charging-discharging capability [4], specific capacitance, flexibility [5] and various electrochemical performances [6]. Electrode preparation and device configuration have also recently been given prior attention. Surface analysis, electrochemical performance, electrical properties and other related properties can be measured to characterize the performance of supercapacitors.

Due to climate change and the depletion of fossil fuels, the development of sustainable and environmentally friendly energy resources has become a necessity. The world requires a stable means of energy storage in electrochemical form. In addition, due to the current development of portable electronics, there is an urgent need to develop high-performance, lightweight and flexible energy storage devices [7]. Supercapacitors are the best candidate for storing electrochemical energy that can be used in wearable electronics [8]. Supercapacitors can directly store electrical energy in the form of static double-layer capacitance and electrochemical pseudocapacitance, which also gives them the advantages of being processable, clean, and applicable in various applications. However, supercapacitors are only suitable for small-scale energy storage applications [9].

The storage ability of supercapacitors is determined by their electrode components. These electrode components have been extensively applied as intrinsically-conductive polymers [10], graphene-conductive polymer composites [11], carbon nanotube (CNT)-conductive polymers [12], melanin based [13], natural cellulose-based hydrogels [14], conductive polymer hydrogels [15] and various metal oxides [16,17], among other things. Conductivity plays a very important role in every aspect of electrodes, such as in wearable electronics [18] and electrochemical applications [19]. Most recently, carbon materials have been used as electrode materials in supercapacitor applications [20]. Carbon-based materials have shown dynamic progress in supercapacitor production [21], providing a capacitance value of 1730 F g^−1^ at 1 A g^−1^ in 1 mol L^−1^ KOH [22], 245 F/g for a three-electrode system [23], 118 F g^−1^ at 1 mA cm^−2^ [24], 300 F g^−1^ in 6.0 M KOH electrolytes [25], and 420 F g^−1^ at 1 A g^−1^ [26]. This indicates the excellent electrochemical performance of cellulose-based carbon materials for the production of supercapacitor materials. Furthermore, wood/cellulose-based carbons have demonstrated the permissible electrical conductivity [27,28]. However, these carbon materials are usually obtained from either graphite [29] or fossil fuels. In order to the reduce environmental contamination caused by traditional processes, scholars have carried out investigations into the easy and sustainable development of electrodes from banana waste. Even though bio-based materials have been extensively studied, extraction, large scale production, cost and homogeneity still remain a challenge [30].

Considering the easy availability and sustainability of banana waste, we have proposed a simple and environmentally friendly conversion of banana peel waste into carbon scaffolds, which can be used as electrode materials for the development of high-performance supercapacitors. These can then be used as energy storage devices in flexible, wearable, lightweight textile electronics. However, the results of our study showed a decreased performance compared to the results cited in the literature, suggesting further exploration is needed.

## 2. Materials and Methods

### 2.1. Carbonization

**Figure 1:**
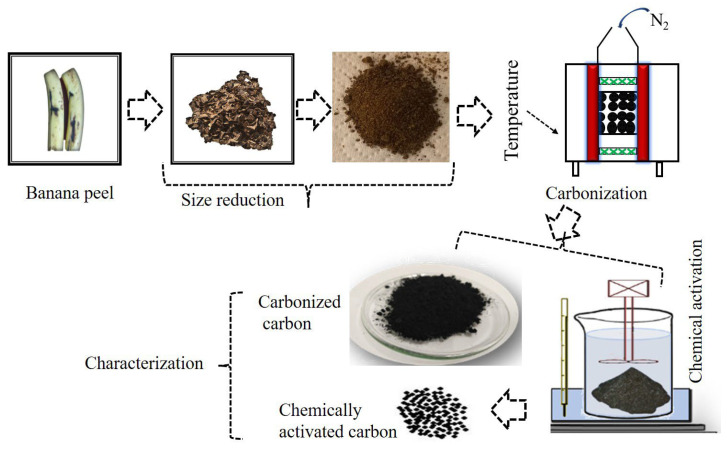
Illustration showing how carbon is obtained from banana peel.

Banana waste (peels) was collected, washed with deionized water (to remove the impurities) and sun-dried after being cut into rectangular pieces, without further treatment, until completely dry. The dried pieces were then downsized with a grinder and then finely grinded into a nano-sized scale. The nano-sized/finely grinded banana peel was inserted into a muffle furnace (thermolyne- F-48000, Dubuque, IA, USA) and heated at 700 °C for 2 h with continuous nitrogen gas flow to convert the banana peel into activated carbon. The general procedure is shown in Figure 1.

### 2.2. Chemical Activation

After carbonization, the charcoal was chemically activated using KOH (charcoal-KOH ratio of 1:3 *w*/*w*). The chemical activation was performed by gently shaking using a mechanical shaker (Heidolph unimax-HU2010, Schwabach, Germany) and subsequently dried using a convection oven (Bernareggio-MF40-VF, Bernareggio, Italy). The charcoal mixed with KOH was left overnight at room temp and dried at 110 °C for 24 h, washed with HCL (0.2 N), rinsed with hot water until the pH became neutral, and then dried at 110 °C for the final product.

### 2.3. Characterization

Fourier transform infrared (FTIR) spectroscopy (JASCO- model-6600, Pfungstadt, Germany) was used to investigate the structural shift and functional groups of the carbonized char and control samples. Thermogravimetric analysis of the samples was carried out in the nitrogen atmosphere using TGA (BJHENVEN, HCT-1), with temperatures ranging from 20 to 700 °C at a rate of 10 °C per minute in a nitrogen atmosphere. Dynamic light scattering (DLS) measurements of the distribution of particle size and the zeta potential of the prepared samples were calculated using size analyzer Zetasizer ver. 7.12 (SN: MAL1149420) from Malvern Instruments Ltd., Worcestershire, UK. 

The pore size and the surface area of the samples was measured and analyzed using the Brunauer–Emmett–Teller (BET) (Quanta chrome-Nova-NOVA4000e) analyzer under a nitrogen atmosphere. The sum of the nitrogen adsorbed at a relative pressure (P/P0) of 0.97 was used to measure the basic surface areas and pore volumes of nitrogen adsorption desorption isotherms determined by a surface area analyzer. 

To investigate the electrochemical performance of the prepared samples, cyclic voltammetry (CV) curves were obtained. The CV analysis were carried out with a BAS 100A (Bioanalytical systems, West Lafayette, IN, USA) electro-analyzer, which was connected to a Dell computer of Pentium 4 model. This was used together with a mercury film working electrode, Ag/AgCl (3 M KCl) reference electrode, and a platinum wire auxiliary electrode. All cyclic voltammograms were recorded at room temperature and the solutions were deaerated with pure nitrogen for 10 min before the voltametric runs and kept over the surface during the measurements. 

Finally, X-ray diffraction (XRD) (XRD-7000, SHIMADZU Corporation, Tokyo, Japan) equipped with a Cu target for generating a Cu Kα radiation with λ = 0.15406 nm and recorded in the range from 10 °C to 80 °C) was measured on an X-ray diffractometer to determine the crystalline phases formed in banana peel activated charcoal at a voltage of 40.0 (kV) and current of 30.0 (mA) with continuous scanning mode.

## 3. Results

**Figure 2:**
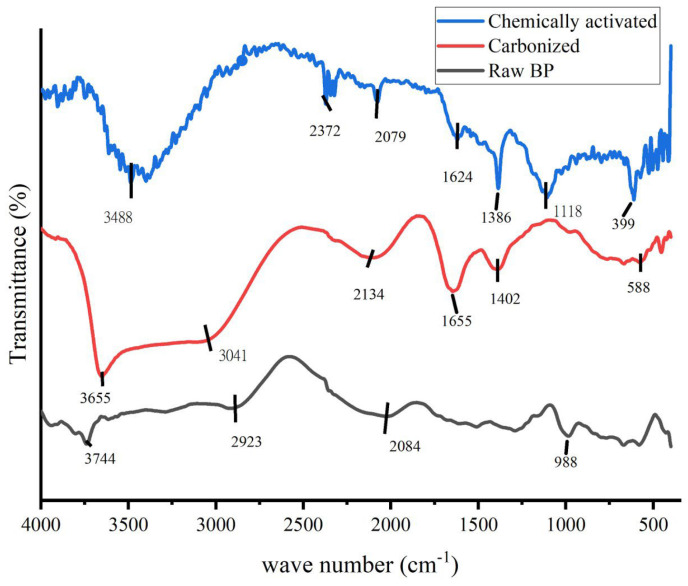
FTIR spectrum of raw banana peel, carbonized banana peel and chemically activated carbonized banana peel.

FTIR is an essential tool and vital characterization practice to explicate the structure of materials at the molecular level. The chemical constituents in terms of bonding arrangement and some information on the chemical components can be obtained through FTIR, as claimed by Bhargava et al. [31]. The FTIR analysis of raw, carbonized and activated banana peel is presented in Figure 2. The presence of different functional groups before (carbonized) raw BP and KOH were activated was confirmed by the FTIR measurements.

In order to understand the nature of the carbonized banana peel and to observe the difference between the carbonized charcoal and the control sample, FTIR spectra (Figure 2) were obtained. As displayed in the graph, differences have been observed and the spectra show the complex nature of the banana peel. Bands appearing at around 3700, 1500 and 1200 cm^−1^ are assigned to O-H stretching and COO- and O-H bending, respectively [32,33]. The significant increase in the intensity of OH stretching is due to additional OH starching and bending because of the activation chemical KOH. During carbonization, the lignin content might be removed. The broad peeks around 3488 cm^−1^ and 1386 cm^−1^ for the KOH-activated sample could probably be the vibration patterns of K_2_CO_3_, which was formed during the reaction with a carbonate of carbonized banana peel (6KOH + 2C ↔ 3H_2_ + 2K_2_CO_3_ + 2K) [34].

**Figure 3:**
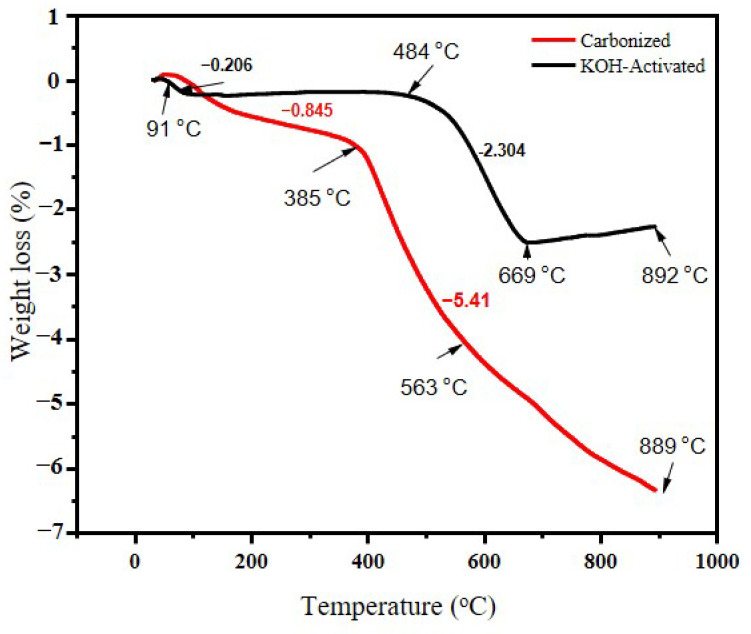
TGA curves for banana peel for carbonized and chemically activated samples at 700 °C.

A thermogravimetric analysis of carbonized banana peel biomass waste was conducted and the rate of weight loss at a given temperature range is illustrated in Figure 3. TGA was applied to determine the carbonization temperature of the waste banana peel. Both samples started to lose mass at about 150 °C and the total weight loss started to separate at around 170 °C. The reason for this could be attributed mainly to the loss of water weight and volatile materials [35]. The high amount of weight loss could also be due to the decomposition of cellulose and hemicellulose [36]. The activated sample continued without much mass loss until 500 °C, when it started to lose mass again. One reason for this could be that chemical activation could help in removing some lignin-based substances that might be subjected to mass loss during heat application.

The mass losses for the carbonized samples began to decrease at around 170 °C and the total weight rapidly decreased until 500 °C. A previous study [37] showed that banana peel loses 90% of its weight at 500 °C, therefore this study intended to set the carbonization temperature at 700 °C. Chemical activation helps in increasing the pore sizes [37] and removes some lignin content, as claimed by authors in Ref. [38]. About 89% of lignin and 79% of extractives can be removed after KOH pretreatment. During the TGA analysis, the weight loss for the carbonized sample was faster than that of the KOH-activated sample.

**Figure 4:**
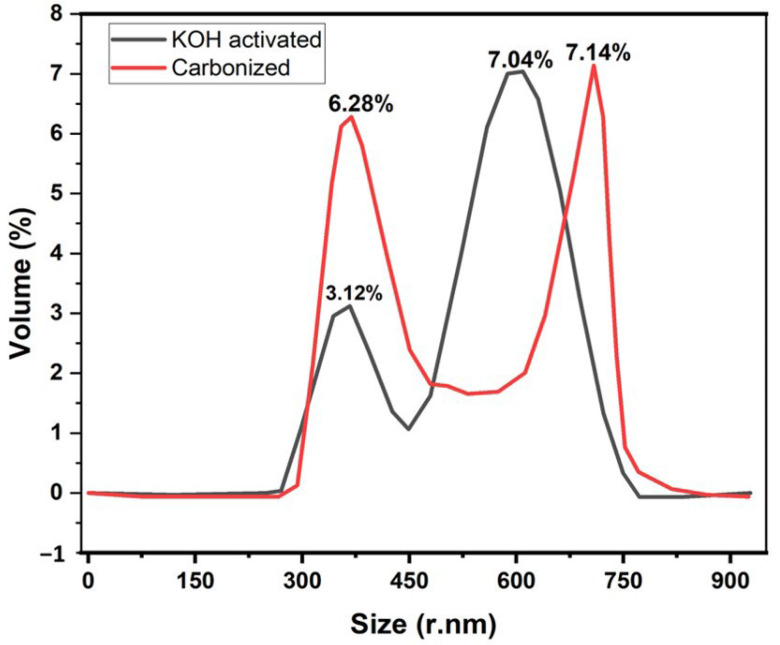
Effect of KOH activation on a size distribution for banana peel carbon.

Figure 4 illustrates the dynamic light scattering of the samples to determine the size distribution of the banana carbon particles. The chemical activation process will help increase the carbon yield and upturns the pore structure [39].

Definition of terms in DLS measurement:

- RI: refractive index, which plays an important role in Mie scattering (how spherical particles of all sizes and optical properties scatter light).
- Z-average size: The intensity-weighted average diameter resulting from the cumulants analysis.

DLS was used to determine the carbonized and the KOH-activated particle size distribution of the nanoparticles dispersed in water with viscosity (cP):0.8872 and dispersant RI: 1.330. According to these DLS-measurements, two strong peaks were observed for KOH-activated samples when compared to the carbonized sample. The results are depicted in Figure 4.

For the samples presented in Figure 4, Z-Average (r. nm): 74.26, maximum volume % (52.4) at size of 4.8 ± 32.62 r. nm for carbonized and Z-Average (r. nm): 53.45, maximum % volume (65.8) at size of 47.97 ± 23.01 r. nm for activated KOH were measured with DLS. Furthermore, another big % volume peak of 48.7 at size of 95.20 ± 29.52 r. nm was observed for carbonized samples at volume when compared to 4.38 ± 23.01 r. nm at 18.14% volume for the KOH-activated sample. It has been proven that the Z-average size increases as the particle size increases, and as particles size increases, the surface area of the particle decreases. Here, we can observe that when charcoal is treated with KOH, the Z-average decreases, which means the sample particles size decreases. Therefore, the KOH-activated sample obtained a high surface area. It was also proved by BET analysis and similar findings reported here [40] that smaller size nanoparticles could possess a higher surface area. Therefore, the shift to high peak in % volume (Figure 4) when treated by KOH could probably be due to the decrease in particle size distribution. 

**Figure 5:**
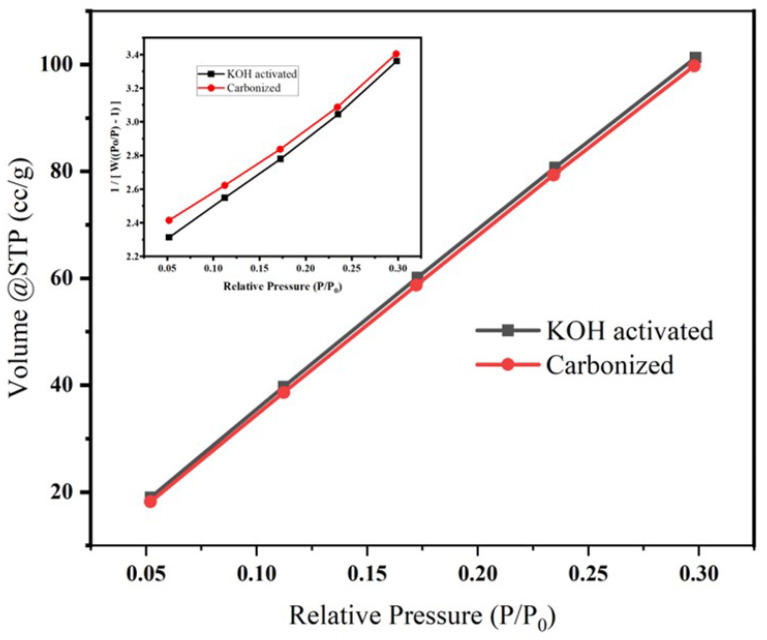
Effect of KOH activation on the pore volume, surface area and pore radius of banana peel carbon. The insert is the corresponding pore size distribution.

A surface area analyzer (Quanta chrome-Nova-NOVA4000e) was used to determine the BET surface of carbonized and activated banana peel. BET surface analysis was carried out on N_2_ adsorption/desorption isotherm at 77.3 K, as shown in Figure 5, and the corresponding isotherms and multipoint BET plot (insert graph in Figure 5) revealed that the surface area of the carbonized and chemically activated samples are 553.862 m^2^/g and 565.024 m^2^/g, respectively. This increase in surface area might be due the impurity differences between the two samples [41]. The increase in surface area might be attributed to the increase in porosity during activation of cellulosed-based materials using KOH [34].

The cumulative pore volume and pore radius (HK method) were found to be 8.05e^−02^ cc/g, 8.237e^−02^ cc/g, 9.237Å and 9.237Å for KOH-activated and carbonized samples, respectively. The Horváth–Kawazoe (HK) method was developed for defining the pore size distribution of materials and states that pores of a given width will fill at a particular relative pressure.

**Figure 6:**
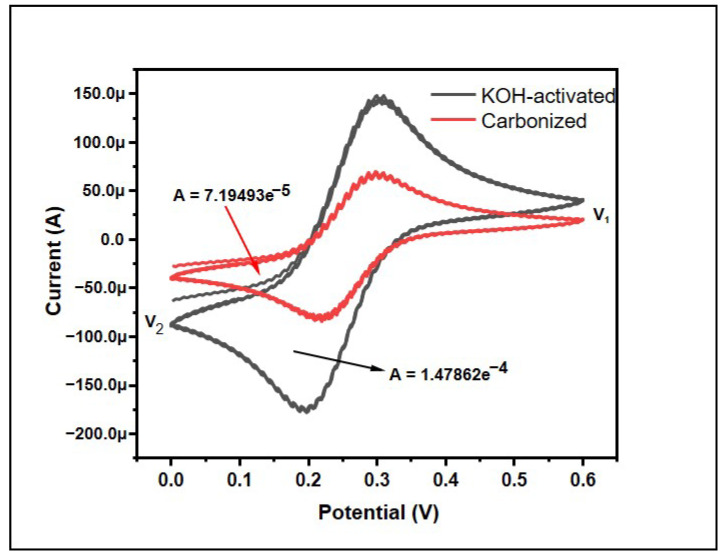
Effect of KOH-activation on cyclic voltammetry results for banana peel carbons.

Cyclic voltammetry (CV) is an effective and accepted electrochemical technique frequently used to study the reduction and oxidation processes of molecular species. The behavior of the samples was also observed using cyclic voltammetry (CV) (Figure 6). The CV curve does not exactly exhibit a comparable quasi-rectangular shape, which is typical for an electric double layer capacitor [42]. This means the banana peel carbon does not exactly possess an electric double layer capacitor but it does have similar features, theoretically. However, the CV is not symmetrical, hence revealing that the electrochemical reaction is largely reversible [43].

The specific capacity was calculated using origin software using the following equations:

(1) $$Cp=\frac{A}{\left[2*mk\;\left(V_{1}-V_{2}\right)*10^{-3}\right]}$$

where *Cp* = specific capacitance in F/g, *A* = the area in CV graph, *m* = mass of the materials in gram, *k* = scan rate in mV/s, and *V*_1_ − *V*_2_ is the potential window in the CV graph. The scan rate of the machine was 50 mV/s and the mass of the materials taken was 3 mg. By using this, the specific capacitance was found to be 0.3997 Fg^−1^ and 0.821 Fg^−1^, which shows the supercapacitor ability of the bio-waste. Furthermore, KOH activation helped to double the specific capacitance of the materials, which could be attributed to the increase in the surface area due to chemical activation.

**Figure 7:**
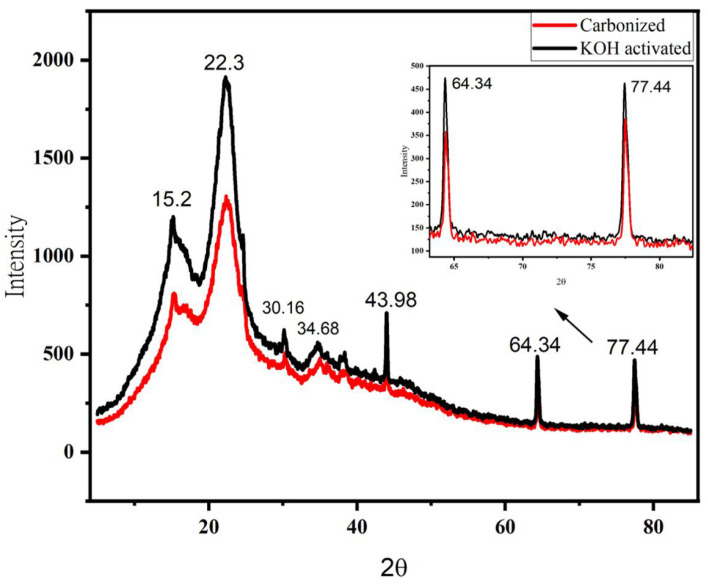
X-ray diffraction (XRD) spectra of carbonized and chemically activated banana peel biowaste samples. The inset is the expanded picture between 2θ of 60° and 85° to show the difference between the two samples. Furthermore, the highest peak at around 2θ = 22.3° could be due to the presence of carbon [45].

The structural investigation of carbon material was analyzed by XRD (Figure 7). The analysis was made at a scan range of 5–85, with a sampling pitch of 0.00200 (degree) and speed of 2.00 (deg/min). The diffraction peaks for the KOH-activated sample are higher than that of the carbonized sample. The highest diffraction peak for the samples is 1958 and 1358 at ~22.3° for KOH activated and carbonized samples, respectively. The lower diffraction peak means a higher degree of amorphous carbon, which means chemical activation helps to develop more pores on the sample [44].

The biochar curve showed less peaks, suggesting that most of the amorphous carbon structure does not exist and revealing that 700 °C is enough to pyrolysis the banana peel. The graphite carbon demonstrated a more intense peak at 22.8° [42]. Two wide diffraction patterns were found at 22.3° and 43.98°, which links to the (002) and (100) reflection planes of graphite (pattern diffraction file data base, PDF 41-1487) [46].

It is believed to be the formation of H_2_, K, K_2_CO_3_ and K_2_O when KOH is reacted during the activation process [47]. It has been reported that KOH was completely converted into K_2_CO_3_ and K_2_O above 600 °C somewhere else [48]. Diffraction peaks for carbonized and KOH activated samples are shown in the same degree range, but not their intensity. The peaks at 2θ = 22.3° (characteristics peaks of graphite 002 [49]), 30.16°, 34.14°, and 43.68° (characteristics peaks of graphite 100 [49]) could probably be due to K_2_O, K_2_CO_3_ [50]. The minor peaks at 2θ = 15.2° indicate the existence of polymorphs of graphene-like nanostructures [51]. The higher intensity occurred with respect to the KOH-activated sample, which is probably be due to the increase in amorphicity when treated with KOH [52]. Furthermore, the crystallinity of the charcoal decreased when treated by KOH [51]. Moreover, the porous surface of the particle is responsible for supercapacitor performance [39]. Hence, the absorption capacity of the treated carbon is high.

The diffraction peaks at 2θ = 64.34° and 77.44° are indexed to the plane’s highest degree of amorphous carbon at this region of activation temperature [42]. However, the higher diffraction peaks disappeared when the 2Tetha increased and both samples revealed broadened peaks, apart from those indicated. The disappearance of the diffraction peaks at a higher degree indicates higher disorder in the samples, which means a higher degree of amorphous carbon state is observed. Based on the results obtained, chemically-activated carbonized banana peel biowaste exhibits the best properties, as it acquires a high amorphous area, which is best for storage purposes.

## 4. Conclusions

In summary, banana peel was successfully valorized by carbonization and chemical activation using KOH. We demonstrated synthesis of high-surface-area activated carbon form the pyrolysis of banana peel waste using KOH activation and carbonization. Chemical activation had a significant effect on morphology (surface area) and cyclic voltammetry results. The surface area of the chemically activated sample was increased by 2.02%. Furthermore, the diffraction pattern changed from 1332 to 1958 at 22.78° from carbonized to chemically activated samples. The result suggested that chemical activation has significant potential for energy storage applications. We anticipate that carbon materials generated from this technique will surely be promising candidates for the production of flexible supercapacitors for energy storage used in smart textile applications.

## Data Availability

Not applicable.
